# Reversed Phi and the “Phenomenal Phenomena” Revisited

**DOI:** 10.1177/2041669519856906

**Published:** 2019-07-26

**Authors:** Brian Rogers, Stuart Anstis, Hiroshi Ashida, Akiyoshi Kitaoka

**Affiliations:** Department of Experimental Psychology, University of Oxford, Oxford, UK; Department of Psychology, University of California San Diego, La Jolla, CA, USA; Graduate School of Letters, Kyoto University, Kyoto, Japan; Department of Psychology, Ritsumeikan University, Kyoto, Japan

**Keywords:** apparent motion, stereopsis, Vernier alignment, reversed phi, phenomenal phenomena, Fraser-Wilcox illusion

## Abstract

Reversed apparent motion (or reversed phi) can be seen during a continuous dissolve between a positive and a spatially shifted negative version of the same image. Similar reversed effects can be seen in stereo when positive and spatially shifted negative images are presented separately to the two eyes or in a Vernier alignment task when the two images are juxtaposed one above the other. Gregory and Heard reported similar effects that they called “phenomenal phenomena.” Here, we investigate the similarities between these different effects and put forward a simple, spatial-smoothing explanation that can account for both the direction and magnitude of the reversed effects in the motion, stereo and Vernier domains. In addition, we consider whether the striking motion effects seen when viewing Kitaoka’s colour-dependent Fraser-Wilcox figures are related to the reversed phi illusion, given the similarity of the luminance profiles.

## Introduction

**Figure 1.**
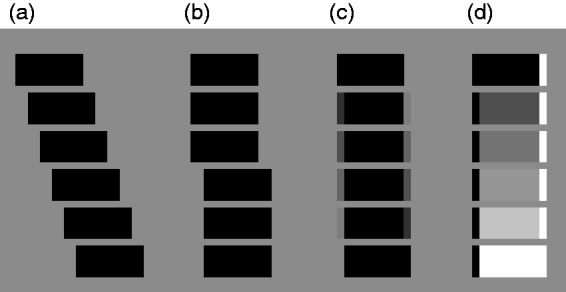
Six successive movie frame showing: (a) smooth, real movement; (b) apparent movement, in which the stimulus jumps from a first to a second position; (c) apparent movement dissolve. The black bar is perceived as moving smoothly to the right; (d) reversed phi dissolve from a black bar to a white bar that is displaced to the right. Apparent movement is perceived to the *left*, opposite to the physical displacement.

An object in *real motion* moves smoothly through a series of positions. An object in *apparent* or *stroboscopic motion* jumps through a series of discrete positions that can easily be rendered in a sequence of movie frames. On the other hand, during an *apparent motion dissolve,* the successive stimuli linger on the screen so that they overlap in time, with the stimulus in frame *n* fading down as the stimulus in frame *n* + 1 fades up. These forms of motion are graphed in Figure 1(a) to (d). The strength of the perceived motion and the magnitude of the perceived displacement during a dissolve between a pair of images is often much greater than that seen during a simple cut.

In 1970, Anstis observed that, during a *dissolve* between an image and a displaced and contrast-reversed version, the perception is of *backward* motion, in a direction opposite to the physical displacement between the two images. He dubbed this effect “reversed phi” or reversed apparent motion (Anstis, 1970; Anstis & Rogers, 1975, 1986; Anstis, Smith, & Mather 2000). This phenomenon is seen only for small displacements (<10 arc min) between the initial and the displaced, contrast-reversed version and, paradoxically, the smaller the physical shift, the greater the perceived backward motion. In 1983, Gregory and Heard reported related effects that they called “phenomenal phenomena.” Many of Art Shapiro's beautiful motion effects (Shapiro, Charles, & Shear-Heyman, 2005) and some of Kitaoka's illusory effects (seen with static patterns; Kitaoka, 2006, 2014) use stimuli that are related to those used to demonstrate the reversed phi effect.

### The Wagon Wheel Illusion

What is the explanation of the reversed phi effect? One possibility is that it is a variant of the well-known wagon-wheel illusion in which the spokes of a wheel are seen to rotate in the opposite direction to their physical motion when a continuously rotating wheel is filmed with a camera that takes a series of discrete frames.

**Figure 2.**
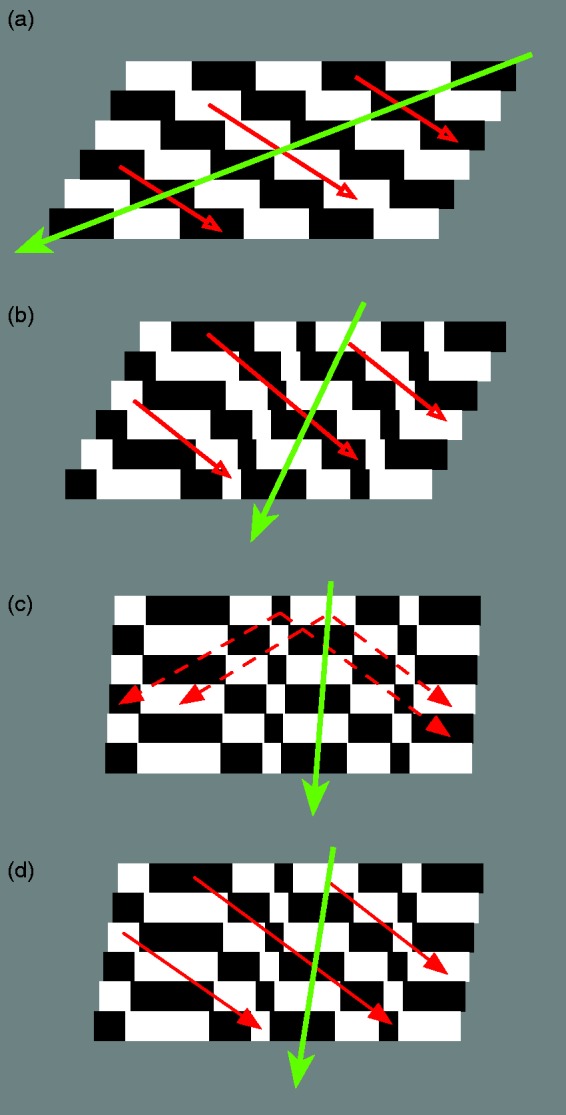
The wagon-wheel illusion. (a) When a repetitive pattern of light and dark bars is displaced to the *left* in successive images but with jumps that have a magnitude greater than half a spatial period (green arrow), the nearest neighbouring light (or dark) bar is in the opposite direction, that is, to the *right* (red arrows). The same thing happens when a repetitive pattern is displaced by a smaller amount to the *left* but reverses in contrast between each presentation. (b) If a pattern has a luminance profile with different width light and dark bars and it displaces to the *left* (green arrow), the nearest neighbouring light (or dark) bar in the contrast-reversed image is also in the opposite direction, that is, to the *right* (red arrows). (c) When the displacement between the pattern and the contrast-reversed version is very small, the nearest neighbouring light (or dark) bar is also to the right (with an amplitude close to half an average spatial period), but there is considerable ambiguity about the direction of displacement (dashed red arrows). (d) If the displacement to the left is increased, the directional ambiguity is reduced.

The wagon-wheel illusion is a form of aliasing in a spatially repetitive moving stimulus when it is sampled at discrete points in time (Figure 2(a)). The perceived reversed direction of motion is present in the sequence of images reaching the eye and therefore tells us little about the properties of the visual system. Like mirages and moire fringes, the wagon-wheel illusion is a consequence of the particular stimuli reaching the eye rather than of the visual system.

Should reversed phi be considered to be a variant of the wagon-wheel illusion? Clearly, if the initial image is a repetitive grating pattern of light and dark bars and the final image is a displaced, contrast-reversed version, the direction of the perceived motion depends on the magnitude of the displacement—in a reversed direction if the displacement is less than half a spatial period and in a forward direction if the displacement is between a half and one spatial period. In other words, the perceived direction of motion is between a given light (or dark) bar and its nearest neighbour of the same contrast (Figure 2(a)). Once again, the perceived motion is in the direction of the physical stimulus reaching the eye and therefore tells us little about the properties of the visual system.

The situation becomes slightly more complicated if the initial image is not a simple repetitive grating pattern but instead is the sort of luminance profile created by a slice through a random dot pattern (Figure 2(b)). In this case, there is not an exact correspondence between the luminance profiles of the initial and the displaced, contrast-reversed version but, for small displacements, there is always a closer match between a given light (or dark) bar in the initial image and its nearest neighbouring light (or dark) bar in the final, contrast-reversed image. Presenting the stimuli as a space-time diagram reveals the reversed direction of the motion in a sequence of images. It is clear that there is motion energy in the reversed direction. Is this all there is to reversed phi? Figure 2(c) and (d) shows that the magnitude of the displacement between the initial and the displaced, contrast-reversed version varies as a function of the *phase shift* of the average spatial period. A small phase shift between the initial image and contrast-reversed version generates the largest average displacement between the nearest neighbouring bars of the same luminance.

How adequate is a wagon-wheel explanation of the reversed phi effect? Anstis and Rogers (1975) reported that the reversed phi effect involving a dissolve from an initial image to a displaced, contrast-reversed version is maximal for very small displacements (a few arc min), and there was little or no reversed motion when the displacement exceeded ∼10 arc min. The wagon-wheel model predicts that the amplitude of the perceived motion in the reversed direction should be maximal with the smallest displacements—approaching half the average spatial period (Figure 2(c)) but does not predict the absence of reversed motion when the displacement exceeds more than 10 arc min.

### A Spatial-Smoothing Explanation

At the time, four observations made us doubt a simple wagon-wheel explanation of the reversed phi effect. First, the reversed phi effect using a temporal *dissolve* is much more powerful than a straight cut from an image to its displaced, contrast-reversed version. Second, reversed phi can be seen when the initial image is a single, dark-to-light luminance *edge* rather than a repetitive or pseudo-repetitive pattern. Third, the perceived amplitude of the reversed phi effect is larger when the stimuli are presented in peripheral vision. Fourth, we discovered similar reversed effects in judgements of disparity and Vernier acuity (Anstis & Rogers, 1975). This led us to propose an explanation of all three effects that was based on the spatial summation or low-pass filtering of the luminance profiles occurring *prior* to the processes responsible for detecting motion, disparity and Vernier alignment (Rogers & Anstis, 1975).

### Reversed Phi

**Figure 3.**
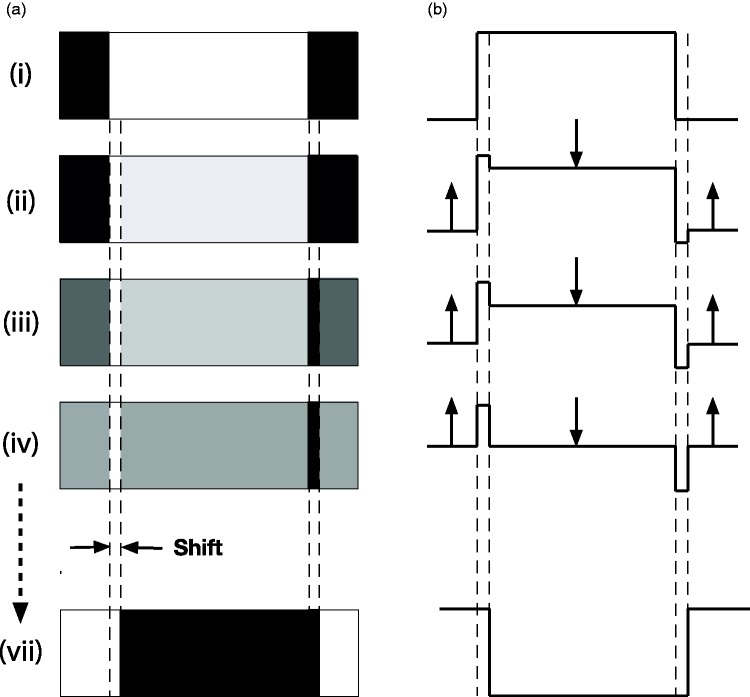
(a) A “reversed phi” sequence of images depicting a continuous dissolve between a light bar on a black background (i) and a contrast-reversed, dark bar shifted slightly to the right (vii). (b) The luminance profiles of the images, and their changes over time, are shown on the right. Note that the widths of the strips are deliberately exaggerated. The reversed phi effect is seen only when the light and dark strips subtend <10 arc min.

Consider a luminance profile that consists of a single broad, white bar on a dark background (Figure 3(i)) and how it changes during a steady and continuous dissolve to a contrast-reversed version that is displaced to the *right* (Figure 3(vii)). The displacement amplitude is small compared to the bar width. Note that the luminance of the surrounding, dark background steadily increases and the luminance of the wide white bar in the centre steadily decreases over time. At the same time, the luminance of a narrow strip at the left-hand edge of the bar remains unchanged (white), while the luminance of a narrow strip at the right-hand edge of the bar remains unchanged (black). These narrow strips turn out to be very important for both the reversed phi effects and for Gregory and Heard’s phenomenal phenomena.

**Figure 4.**
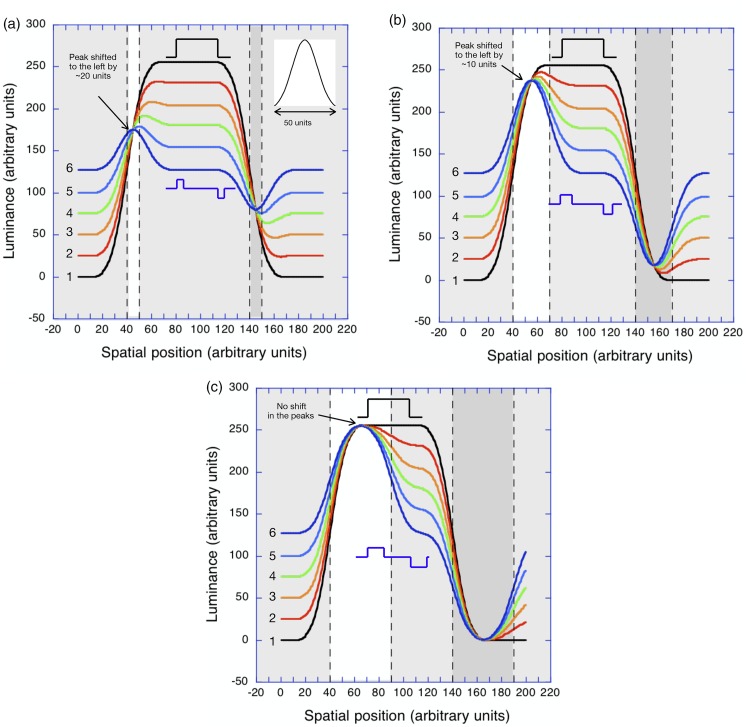
The results of modelling the first six stages of a reversed phi dissolve between a light bar on a black background and a contrast-reversed, dark bar shifted slightly to the *right* when the luminance profiles are smoothed by the low-pass Gaussian filter shown in the upper right. (a) When the displacement is small (10 units), the peaks and troughs of the smoothed profile shift progressively to the *left*. (b) When the displacement is increased (30 units), the peaks and troughs of the smoothed profiles still shift to the left but by a smaller amount. (c) When the displacement is increased farther, (50 units) the peaks and troughs show *no* shift and the zero-crossings of the major contours all line up at the boundary between the grey surround and the light strip.

Figure 4(a) shows what happens when a similar luminance profile with a strip width of 10 (arbitrary) units is smoothed by a low-pass Gaussian filter (inset) with a diameter of 50 (arbitrary) units. The different coloured lines (black through to dark blue), which are smoothed versions of those in Figure 3(b), represent the first six stages of a continuous dissolve from the initial white bar to a point halfway through the dissolve to the displaced, contrast-reversed dark bar.^1^ The different coloured lines show that there is a significant displacement of the *peak* of the response (corresponding to the light strip) to the *left*—in other words, in the opposite direction to the displacement of the contrast-reversed version (which is to the *right*). On the right-hand side of the central bar, the trough (corresponding to the dark strip) also displaces to the *left*. In addition, Figure 4(a) shows that all the major dark-to-light and light-to-dark contours displace to the *left* during the dissolve. Hence, the spatial summation model correctly predicts the direction of the reversed apparent motion that Anstis reported.

**Figure 5.**
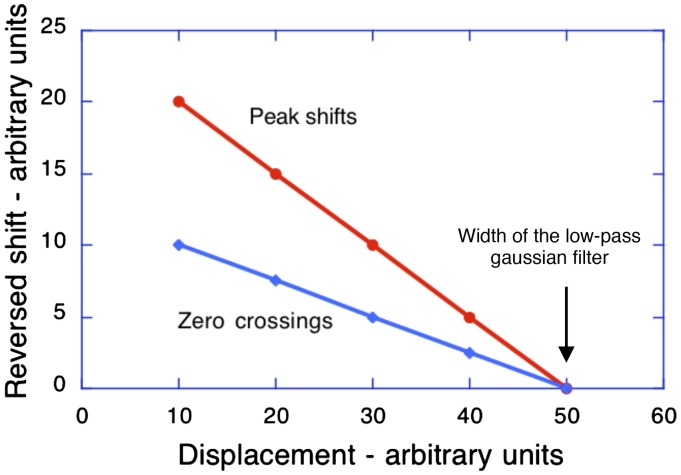
The spatial-smoothing proposal shows how the size of the shifts of both the peaks (and troughs) and the zero-crossings of the major contours decrease with increasing displacement of the contrast-reversed image. The shifts of the peaks and troughs are double those of the zero crossings. The slope of the zero-crossing function is minus 10/40 = −0.25 and the slope of the peak-shift function is minus 20/40 = −0.5.

In Figure 4(b), exactly the same luminance profile is smoothed by the same low-pass Gaussian filter, but the displacement amplitude (strip width) has been increased to 30 (arbitrary) units. In this case, however, the displacements of both the peak of the response (corresponding to the light strip) and the trough of the response (corresponding to the dark strip) during the dissolve are *smaller*. With a displacement of 50 units (Figure 4(c)), neither the peaks nor the troughs are displaced during the stages of the simulated dissolve. Figure 5 shows the size of the displacements of the peaks and zero-crossings of the smoothed luminance profiles during a dissolve between a positive and a displaced negative (Figure 4) as a function of the size of the displacement.

Note that the value of the displacement at which the reversed effect should be no longer seen (i.e., 50 units) is the same as the spatial extent of the low-pass smoothing function and therefore can be used to provide an estimate of the extent of spatial summation at a particular eccentricity and for a particular visual task.

In the graphs shown in Figure 4, note that both the size of the displacement and the size of the smoothing function are expressed in arbitrary units. This means that the *ratio* between the dimensions of luminance profile and the smoothing function is size invariant. In Figures 4(c) and 5, the displacement (50 units) that no longer creates a reversed effect provides an estimate of the extent of spatial summation—that is, 50 units. If the size of the smoothing function is doubled (as is the case for peripheral receptive fields), the perceived amplitude of the reversed phi motion should double and the displacement at which the reversed motion breaks down should also double. This is precisely what Anstis and Rogers (1975) found. When the reversed apparent motion stimuli were presented away from the fovea (to regions where the receptive fields are larger), the amplitude of perceived reversed motion increased and the maximum displacement of the contrast-reversed negative image that still produced reversed motion also increased.

### Reversed Stereo

**Figure 6.**
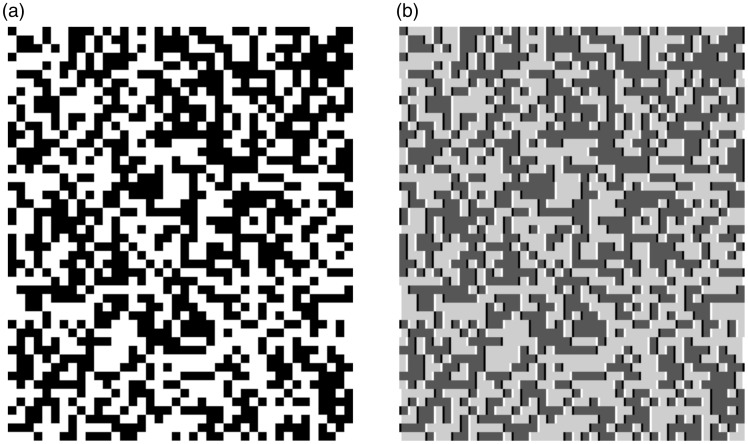
A demonstration of the reversed stereo effect in which observers should perceive a square wave corrugated surface with alternating horizontal bands of crossed and uncrossed disparities. Image (b) is a composite of the original image (a) plus a displaced negative version of that image. In the uppermost corrugation, the displacement of the negative image in the composite image (b) is to the *left* but when the image is spatially smoothed, the effective position of the contours is to the *right*. Hence, with cross-eye fusion, the uppermost band should appear to have a crossed disparity (i.e., lie in front), whereas the second uppermost band should appear to have an uncrossed disparity (i.e., lie behind).

The second piece of evidence that spatial-smoothing is responsible for the reversed phi effect was the discovery of similar effects in the disparity domain (Rogers & Anstis, 1975). It is well known that presenting a random dot pattern to one eye and a contrast-reversed version to the other eye produces binocular rivalry and no impression of depth (Julesz, 1971). Not surprisingly, presenting a single white bar to one eye (Figure 3(i)) and a displaced, contrast-reversed dark bar to the other eye (Figure 3(vii)) yields no impression of depth. However, if a single white bar is presented to one eye (Figure 3(i)) and the other eye is presented with one of the composite images created during a dissolve from the white bar to a displaced, contrast-reversed dark bar (e.g., Figure 3(ii) or (iii)), stereopsis is obtained and the direction of the perceived depth is in the *reversed* direction to the disparity of the contrast-reversed dark bar (Rogers & Anstis, 1975) (Figure 6).

**Figure 7.**
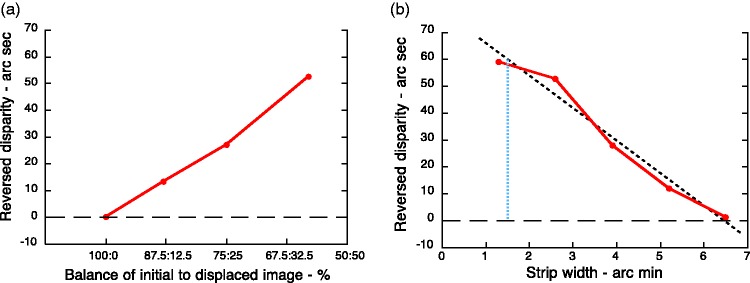
(a) The matched amplitude of the reversed depth increases as the balance between the initial (positive) image and the displaced, contrast-reversed negative version presented to the right eye changed from 100:0 to 60:40 percent. (b) The maximum reversed depth was seen with the smallest displacement (1.3 arc min) and fell steadily to zero when the strip width was increased to 6.5 arc min (Rogers & Anstis, 1975). The slope of the best-fitting straight line (dashed) is minus 60″/(60 × 5′) = −0.2.

Figure 7(a) shows R&A’s results. The amount of reversed depth increases with each subsequent stage of the dissolve, reaching a maximum when the left eye saw the single white bar and the right eye saw a composite consisting of 60% of the original white bar and 40% of displaced, contrast-reversed dark bar (Figure 7(a)). If the right eye saw a composite that contained 50% or more of the displaced, contrast-reversed dark bar, binocular rivalry occurred and stereopsis could no longer be obtained. In other words, as soon as the two eyes saw images that were of opposite contrast (however small), no depth was seen, in line with Julesz’s original observation. Moreover, the reversed stereo effect was strongest when the displaced, contrast-reversed dark bar had a disparity of 1.3 arc min (Figure 7(b)) and the amount of reversed depth fell steadily as the disparity was increased to ∼6.5 arc min, where it was abolished (Rogers & Anstis, 1975). Note the quantitative as well as qualitative similarity between this graph, in which the average gradient is −0.20 (Figure 7(b)), and the predictions of the spatial-smoothing model (Figure 5), in which the gradient is −0.25.

This pattern of results is consistent with the idea that reversed stereo, like reversed phi, can be explained by the simple operation of spatial-smoothing the images before the depth or motion of the displaced contours is extracted. Moreover, modelling the effects of spatial summation (Figures 4 and 5) suggests that the receptive fields involved in the processing of binocular disparities close to the fovea should have summatory centres of ∼6.5 arc min.

### Reversed Vernier Alignment

**Figure 8.**
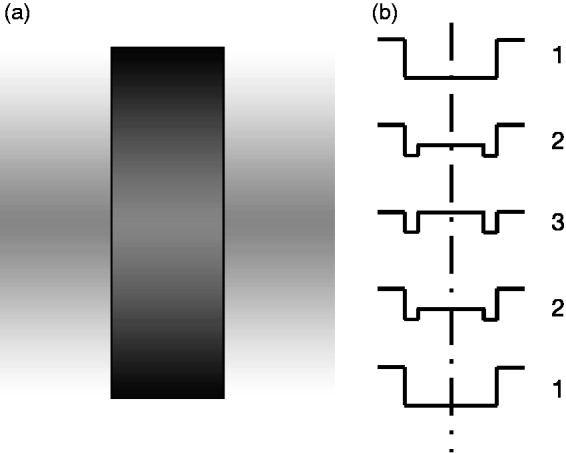
A demonstration of the reversed Vernier offset effect. The upper, left-hand part of the greyscale image shows the first three Stages (1–3) of a dissolve from a light-to-dark edge to a dark-light edge displaced to the right (cf, the right-hand edge of Figure 3). The upper, right-hand part of the image is a mirror-reversed copy. The lower part of the greyscale image shows the three stages of the dissolve back to the original state (3–1). The luminance profiles, deliberately exaggerated in horizontal scale, are shown on the right. The sides of the central rectangle appear to bow *outwards* in the centre, at the point where the luminance of the surround equals that in the centre (3). This is in the opposite direction to that of the displaced negatives in the composite image. The image should be viewed so that the thin black lines subtend <4 arc min.

The third piece of evidence to support the spatial-smoothing explanation was the discovery of a similar effect in the judgement of Vernier alignment (Anstis & Rogers, 1975). The observer’s task in that experiment was to align the boundary of a single, dark-to-light edge (Figure 3(i)) in the upper part of display with an edge that corresponded to one of the stages of a dissolve between a dark-to-light edge and a displaced, contrast-reversed light-to-dark edge in the lower part of the display (e.g., Figure 3(iii)). In other words, the two edges that the observer was asked to align spatially were identical to the images used in a reversed apparent motion sequence over time and the pair of images used in a binocular, reversed stereo presentation. When the composite image was a mixture of the light-to-dark edge and a contrast-reversed edge that was displaced to the *right*, observers judged the composite edge to be displaced to the *left* (see Figure 8).

**Figure 9.**
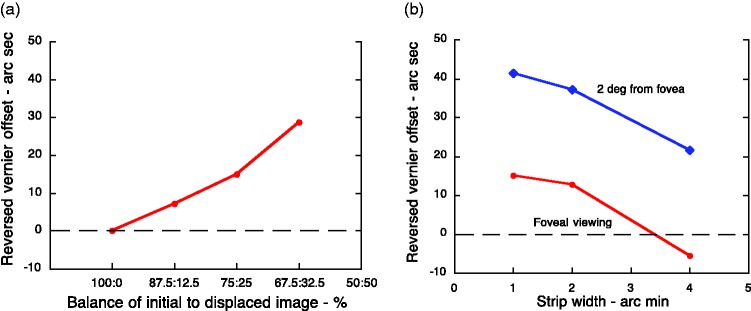
(a) As the balance between the initial (positive) image and the displaced, contrast-reversed negative version in the lower part of the display changed from 100:0 to 67.5:32.5 percent, the amount of reversed Vernier offset *increased* (Perrett, 1976). (b) As the magnitude of the displaced negative (corresponding to the strip width) increased, the amount of reversed Vernier offset *decreased* (Perrett, 1976).

The magnitude of the effect was measured by asking observers to shift the horizontal position of the composite pattern in the lower part of the display until it appeared to be aligned with a simple dark-to-light edge in the upper part of the display (Perrett, 1976). As previously described for reversed apparent motion and reversed stereo, the amount of shift needed for the contours to appear aligned *increased* with the balance of the displaced, contrast-reversed light-to-dark image that was present in the composite (Figure 9(a)). Moreover, the apparent Vernier offset was maximal for very small offsets of the contrast-reversed light-to-dark edge and was abolished when the offset exceeded ∼3.5 arc min (Figure 9(b)). This pattern of results is consistent with that described previously for the reversed motion and reversed stereo effects, although the predicted size of the summatory centres of the receptive fields involved in making Vernier judgments close to the fovea should be smaller at around 3.5 arc min.

Additional support for the spatial-smoothing explanation comes from the finding that the reversed Vernier alignment effects were substantially larger when the display was seen in slightly peripheral vision (Figure 9(b)). It is well-established that receptive fields (including their summatory centres) increase in size with increasing eccentricity. The spatial-smoothing explanation not only predicts that larger displacements of the contrast-reversed image would be tolerated before the effect broke down (Figure 4(c)) but also that the size of the reversed alignment effect would be greater. Figure 9(b) shows the experimental evidence to support both predictions.

The three effects described so far—reversed apparent motion, reversed stereo and reversed Vernier alignment—can all be accounted for by a simple, parsimonious explanation in which the visual system blurs or spatially smooths the physical contours of the stimuli prior to the extraction of motion, stereo or Vernier alignment. Our simulations show that if the stimuli used in the reversed phi, reversed stereo and reversed Vernier effects are spatially smoothed, either optically or neurally, the major contours will be displaced in the reversed direction and hence *all* models of motion, stereo and Vernier alignment must predict the reversed effects, just as they would if the contours were actually displaced in that direction. This is not to say that the spatial-smoothing does not form part of the processes used to extract motion, stereo or Vernier alignment information but rather that the reversed effects themselves do not depend on particular ways in which motion, stereo or Vernier alignment information is extracted.^2^

## Gregory and Heard’s “Phenomenal Phenomena”

**Figure 10.**
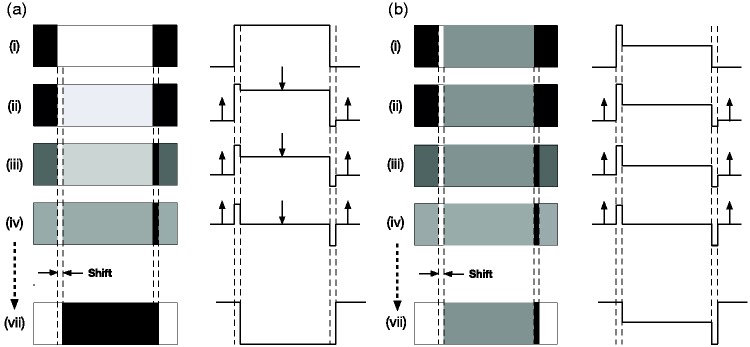
A comparison of the greyscale images and their luminance profiles used in a typical “reversed phi” sequence (a) and those used in Gregory and Heard’s “phenomenal phenomena” (b).

So far, we have shown that the reversed apparent motion, reversed stereo and reversed Vernier alignment effects can all be explained by spatial-smoothing. Can this simple proposal also account for the effects that Gregory and Heard (1983) reported in their paper: “Visual dissociations of movement, position, and stereo: Some phenomenal phenomena”? There are many similarities in the luminance profiles used by G&H and A&R, as Kitaoka (2006) has pointed out previously. Compare the reversed phi luminance profiles in Figure 10(a) with the luminance profiles used by Gregory and Heard (Figure 10(b)) during an apparent motion dissolve. In both cases, the stimuli consist of a thin, light strip on one side of a central rectangle and a thin dark strip on the other side. In addition, the luminance of the (initially) dark surround increases from being iso-luminant with the dark strip to being iso-luminant with the light strip. The only difference in the profile used by Gregory and Heard is that the central rectangle stays at the *same* mid-grey, whereas in the profile used by Anstis and Rogers, the luminance of the central rectangle *decreases* as the luminance of the surround increases. Note that the A&R and the G&H luminance profiles are identical at the midpoint of the dissolve (Figure 10(iv)).

### Spatial-Smoothing

**Figure 11.**
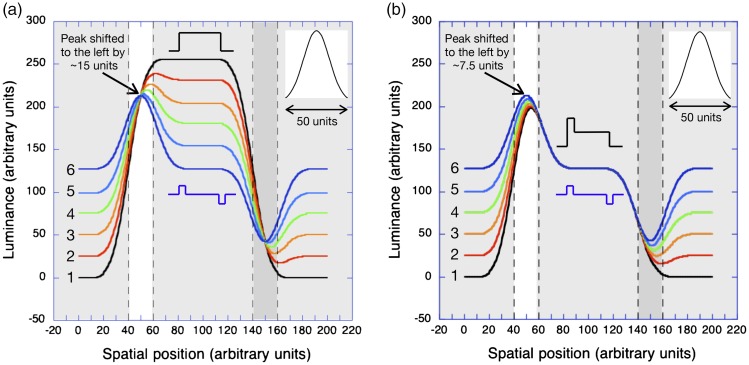
The results of modelling the low-pass filtering of the luminance profiles used in A&R’s experiments (a) and those used in G&H’s experiments (b) with a strip width of 20 units and Gaussian spatial smoothing of 50 units. The different coloured curves represent the first six stages of a dissolve between the initial image (1 = black line) and the halfway point in the dissolve (6 = dark blue line). Note that both the unsmoothed and smoothed luminance profiles at the halfway point in the dissolve are the same in the two studies.

When the R&A profiles are spatially smoothed or low-pass filtered, the smoothed contours shift in the *same*, leftward direction. This is in the *opposite* direction to the location of the rightward-displaced, contrast-reversed image in the case of the reversed phi effect (Figure 11(a)). The smoothed contours of G&H profiles also shift in the *same*, leftward direction: that is, *away* from the central rectangle for the light strip and *towards* the central rectangle for the dark strip, to use G&H’s terminology (Figure 11(b)).

When the width of the narrow strips is small (20 units), the smoothed profile of both R&A’s and G&H’s stimuli show a steady shift in the location of both the light peak (to the left of the rectangle) and the dark trough (to the right). Both move *leftwards*, opposite to the direction of the rightward-displaced, contrast-reversed copy. Figure 11 also reveals that the magnitude of the predicted shift is twice as great (∼15 units) for the A&R stimuli than for the G&H stimuli (∼7.5 units). This is a consequence of the *decrease* in luminance of the central rectangle in A&R’s stimuli (and absent in the G&H stimuli). Moreover, the spatial-smoothing proposal predicts that if the width of the narrow strips in G&H stimuli is increased, the phenomenal phenomena motion should also be abolished.

### Similarities and Differences in the Experimental Results

Empirically, there are many similarities between A&R’s and G&H’s reversed effects. Gregory and Heard reported that the perceived motion in their phenomenal phenomena was largest for small strip widths (their strip width was 1.8 arc min) and disappeared when the width was increased to 10 arc min (consistent with Anstis and Rogers’ observations). Moreover, both studies reported that the reversed motion effects were larger when the stimuli were seen in peripheral vision.

However, whereas Anstis and Rogers reported that the *direction* of all three effects—reversed phi, reversed stereo and reversed Vernier alignment—was in the same (reversed) direction, Gregory and Heard reported significant differences. Indeed, their paper was entitled “Visual dissociations of movement, position and stereo depth: Some phenomenal phenomena.” If our spatial-smoothing model is correct, all three of G&H’s effects should be seen in the *same* (reversed) direction, although the magnitude of the effects is likely to be different because the stimuli used to elicit the effects are slightly different. As a consequence, the findings of Gregory and Heard appear to be at odds with both our results and the spatial-smoothing explanation.

### G&H’s Reversed Motion

The first point to note is that the methods (as well as the stimuli) used by Gregory and Heard to measure the motion, stereo and Vernier effects were different from those of R&A. In the case of G&H’s motion experiment, they measured the amplitude of the perceived reversed motion seen during a back-and-forth dissolve between a pair of the stages shown in Figure 10(b)—for example, between (ii) and (iii)—rather than over the complete sequence from (i) to (vii). However, the perceived motion was always in the *reversed* direction during each of their back-and-forth dissolves and therefore compatible with our experimental findings, as G&H acknowledge (p. 2 of their paper).

### G&H’s Reversed Stereo

The stereo stimuli seen by observers in the R&A experiment consisted of the initial (positive) image (Figure 10(a), (i)) to one eye and a composite (positive-plus-displaced negative) image to the other eye (e.g., Figure 10(a), (ii) or (iii)). The perceived depth in their experiment was in the *opposite* direction to disparity of the displaced negative image. Rivalry, rather than depth, was seen when the percentage of displaced negative in the composite was ≥ 50%—that is, Stages (iv) to (vii).

**Figure 12.**
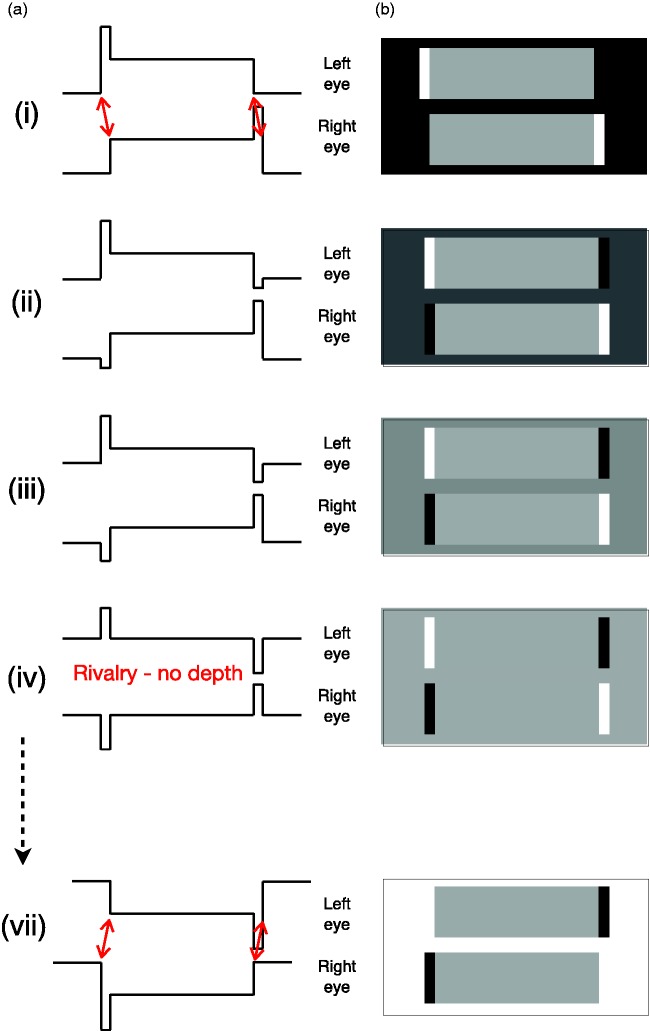
The images (b) and their luminance profiles (a) used in G&H’s reversed stereo experiment. Left-right reversed images were presented to the two eyes. The major contours of the luminance profiles reveal an *uncrossed* disparity in the first image pair (i) and a *crossed* disparity in the last image pair (vii). When the opposite contrast images were presented to the two eyes in (iv), no depth was seen. Note that the widths of the strips are deliberately exaggerated. The reversed stereo effect is seen only when the light and dark strips subtend < 6 arc min.

The stereo stimuli used in G&H experiment consisted of one of the composite profiles shown in Figure 10(b) being presented to one eye while an identical, left-right reversed image was presented to the other eye. When the first image was presented to the left eye and the left-right reversed image to the right eye (Figure 12(i)), the perceived depth was in an *uncrossed* direction by an amount similar to that of the strip width (1.8 arc min). This is not surprising because the luminance profiles show that the major dark-to-light and light-to-dark contours have an uncrossed disparity equal to the strip width (red arrows). Likewise, when the last image was presented to the left eye and the left-right reversed image to the right eye (Figure 12(viii)), the perceived depth was in a *crossed* direction by an amount similar to that of the strip width (1.8 arc min - red arrows).

**Figure 13.**
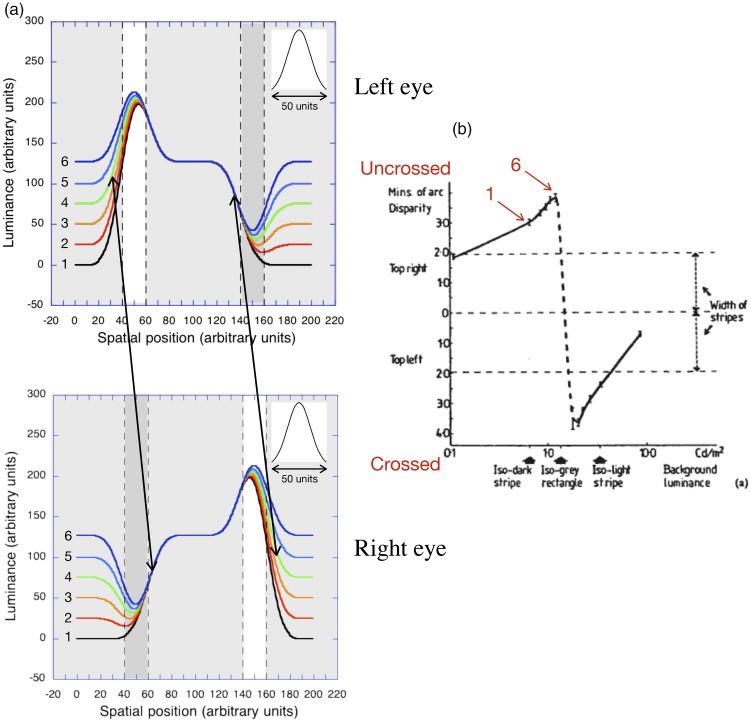
(a) The results of modelling the effects of low-pass filtering G&H’s images presented to the two eyes. The zero-crossings (Z/Cs) of the major dark-to-light and light-to-dark contours in the left eye’s image are shifted progressively to the *left* between Stages 1 (black line) and 6 (dark blue line), creating an *increasing* uncrossed disparity between the eyes. Likewise, the Z/Cs of the matched dark-to-light and light-to-dark contours in the right eye’s image are shifted progressively to the *right* between Stages 1 and 6, also creating an *increasing* uncrossed disparity. (b) G&H’s stereo results showing increasing uncrossed disparity from the iso-dark stripe (1) to the iso-grey rectangle (6).

However, when the intermediate images and their left-right reversed versions were presented stereoscopically to observers (e.g., Figure 12(ii) or (iii)), G&H reported that the amount of perceived depth *increased* in an uncrossed direction (Figure 13(b)). It is not obvious why the perceived depth should change in this way based on the luminance profiles alone, but this result is precisely what the spatial-smoothing model predicts. The smoothed contours in Figure 13(a) show that the major dark-to-light contour in the left eye’s image is displaced progressively to the *left* during the first six stages of the dissolve while the matched dark-to-light contour in the right eye’s image is displaced to the *right* and similarly for the matched light-to-dark contours.

In other words, when the surround is *darker* than central bar, the predicted disparity increases in the *uncrossed* direction. These modelling results match the increase in perceived depth in an *uncrossed* (reversed) direction reported by G&H (Figure 13(b)). At the point when the luminance of the background was the same as that of the central rectangle Figure 12(iv), G&H reported that the depth was not “stable,” and this is not surprising because the corresponding strips in the two eyes have *opposite* contrasts. This finding corresponds to what R&A reported—depth matching broke down when the percentage of displaced negative in the composite image (positive-plus-displaced negative) was ≥50% (Figure 7(a)).

When G&H’s observers viewed the stereo image pairs between the midpoint and the final images of Figure 12(v) to (vii), they reported that the amount of perceived depth decreased from a large *crossed* disparity value before reaching ∼1.8 arc min of crossed disparity when the surround luminance was white (Figure 13(b)). This pattern of results again corresponds to the predictions of our spatial-smoothing model. Once the differences in the methods and stimuli used by G&H are taken into account, there are no differences in the pattern of results in the two studies, and both can be explained in terms of spatial-smoothing of the luminance profiles.

### G&H’s Reversed Vernier Offset

**Figure 14.**
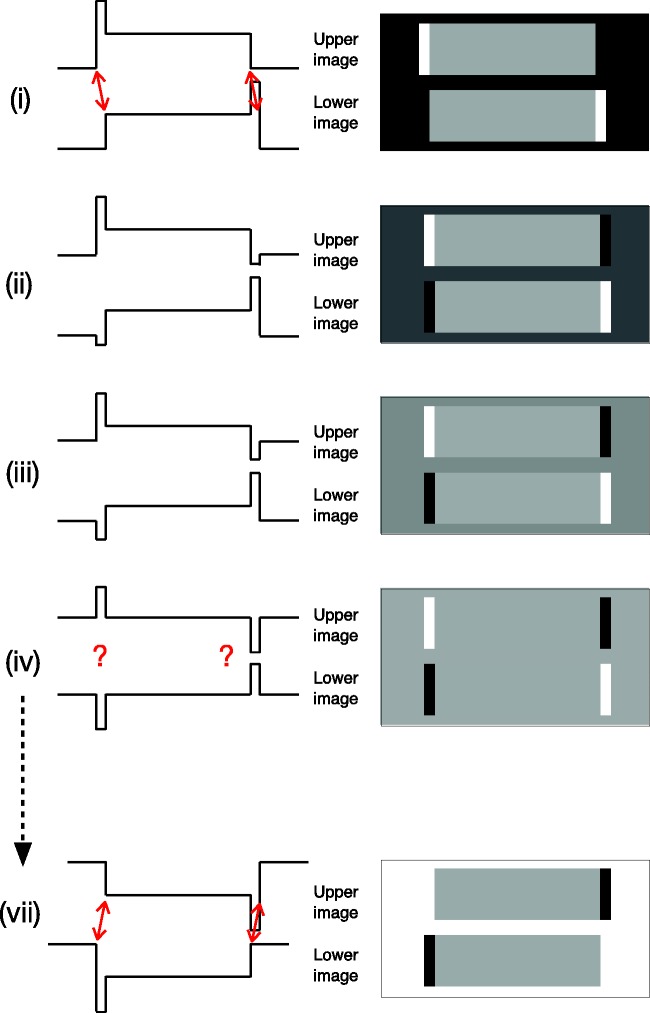
The images (b) and their luminance profiles (a) used in G&H’s Vernier alignment experiment in which the lower image is a left-right reversed version of the upper image. Note that the widths of the strips are deliberately exaggerated—the reversed Vernier effect is seen only when the light and dark strips subtend <4 arc min.

In their paper, Gregory and Heard claimed that the results in their Vernier alignment task showed a “Vernier shift (that) is in the opposite direction to that reported by Anstis and Rogers.” However, we attribute this to the differences in the stimuli they used. The stimuli they used for their Vernier alignment study consisted of a pairs of images, located one above the other, and the observers’ task was to judge and null out any apparent misalignment until “both rectangular figures appeared to be vertically aligned” (p. 2). The pairs of images were same as those used in their stereo experiments (normal and left-right reversed versions) but presented one above the other for their Vernier alignment task (Figure 14).

When the surround had the same luminance as the dark strip (Figure 14(i)), it is not surprising that observers perceived the contours of the upper stimulus to be to the *left* of the lower stimulus because the major dark-to-light and light-to-dark contours of the upper image are displaced by 1.8 arc min to the *left*, relative to the lower image. Similarly, when the background had the same luminance as the light strip (Figure 14(vii)), it is not surprising the observers perceived the contours of the upper stimulus to be to the *right* of the lower stimulus by an amount that corresponded to the width of the narrow strips.

**Figure 15.**
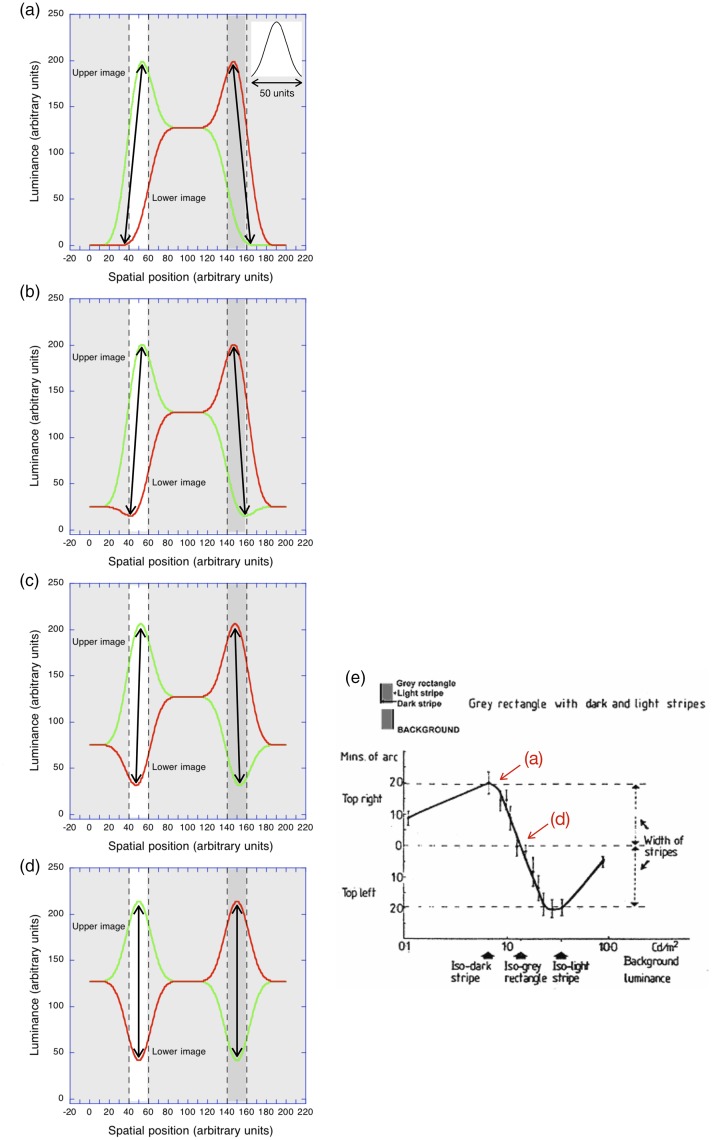
Modelling the effects of low-pass filtering on four of the luminance profiles used in G&H’s Vernier alignment study. The green lines show the smoothed luminance profiles in the *upper* image and the red lines the smoothed luminance profiles in the *lower* image. The arrows show the relative positions of a peak and a trough for each of the stimuli pairs. In (a), the peak (or trough) in the upper image and the trough (or peak) in the lower image are *misaligned*, as shown by the black arrows.^3^ As the pair of images approach the midpoint, the extent of the misalignment decreases in (b) and (c) until the peak (or trough) in the upper image and the trough (or peak) in the lower image are *aligned* in (d). (e) G&H’s Vernier alignment results showing a misalignment at the “iso-dark” point (a) that *decreases* towards alignment at the “iso-grey” point (d).

However, when observers were asked to align the intermediate pairs of images (e.g., Figure 14(ii) or (iii)), G&H reported that the amount of perceived offset was progressively *reduced* (Figure 15(e)) as the background luminance approached the “iso-grey” point where the luminance of the background was the same as that of the central rectangle. At the point when the luminances of the background and the central rectangle were the same (Figure 14(iv)), observers reported no offset between the upper and lower images. The explanation of this particular pattern of results is not obvious from inspection of the luminance profiles themselves (Figure 14).

We can answer this question by considering the responses their observers made to the particular Vernier task depicted in Figure 14(iv). When the same pair of images was presented separately to the two eyes in G&H’s stereo experiment (Figure 12(iv)), observers saw rivalry rather than depth because the corresponding strips in the binocular images were of opposite contrast. However, when the same pair of contours were presented one above the other in a Vernier offset experiment, it was quite possible for observers to align the narrow strips, even though they had opposite contrasts. Hence, there should be little or no Vernier offset. This was the result reported by G&H at the “iso-grey” point (Figure 15(e)). Note that the instruction given to observers was to adjust the horizontal position of the lower figure until “both rectangular figures appeared to be vertically aligned.” When the luminance of the surround was the same as that of the central area (Figure 14(iv)), the grey “rectangle figures” between the light and dark strips were of course aligned.

This explains why their observers saw no Vernier offset in their “iso-grey”, but how can we account for the results reported by G&H when observers were presented with the intermediate stages, for example, 14(ii) or (iii)? If observers were attempting to align the positions of the major contours, the spatial-smoothing model (Figure 15) predicts that the apparent Vernier offset should be *larger* with the composite images 14(ii) and (iii) compared with 14(i). The modelling results shown in Figure 15(a) to (d) show that the horizontal separation between the contours in the upper (green) and lower (red) images *increases* from 15(a) to 15(b) and from 15(b) to (c).

However, if observers were attempting to align the opposite contrast strips (rather than the major contours) in the way they did when presented with the images shown in Figure 14(iv), the spatial-smoothing model predicts that the perceived Vernier offset of the corresponding peaks and troughs should *decrease* (Figure 15(a) to (d)). The horizontal separation between the peak (or trough) in upper (green) profile and the corresponding trough (or peak) in the lower (red) images *decreases* from a maximum in Figure 15(a) to zero in Figure 15(d) where the opposite contrast strips are aligned.

**Figure 16.**
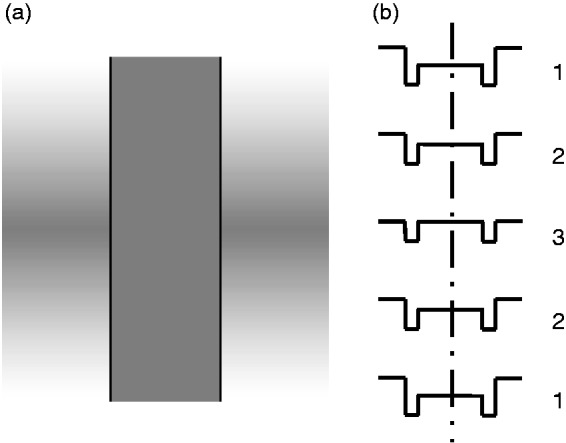
A demonstration of the reversed Vernier offset effect using modified versions of G&H’s stimuli. The sides of the central rectangle appear to bow *outwards* in centre (Stage 3)—when the luminance of the surround is the same as that of the central rectangle (“iso-grey” in G&H’s terminology), that is, in the opposite direction to that of the displaced negative in the composite image. The effect is evident but less pronounced compared with A&R’s stimuli (Figure 8) but more obvious when viewed slightly peripherally. The display should be viewed so that the thin black lines subtend <4 arc min.

As a consequence, the results reported by G&H are consistent with the suggestion that observers were attempting to align the opposite contrast strips in the upper and lower images and therefore perfectly compatible with our own results and with our spatial-smoothing explanation. Moreover, we think it very likely that if G&H had used a pair of their images in their Vernier alignment task that represented different stages of a dissolve between the dark background (iso-dark strip) and their light background (iso-dark strip), rather than left-right reversed versions, they would have obtained similar results to those we reported. Figure 16 provides evidence to support this. There is a similar reversed Vernier alignment effect using a modified version of G&H’s stimuli in which the luminance of the central rectangle remains constant.

Gregory and Heard (1983) argue that their own findings support the idea that different channels or mechanisms are used in the processing of motion, stereo and Vernier alignment and that these differences could account for the different patterns of results they found in their motion and Vernier alignment tasks. We agree that there are likely to be differences between the motion and Vernier alignment channels, but we suggest that the different patterns of results that they observed were not due to channel differences per se but rather a consequence of the instructions in their Vernier alignment task, which encouraged observers to align the opposite contrast strips (Figure 15(a) to (d)) rather than the corresponding light-to-dark (or dark-to-light) edges of the major contours in the two images.

## Conclusions—Reversed Phi and Phenomenal Phenomena

Our conclusion is that the spatial-smoothing explanation proposed by Anstis and Rogers (1975) and Rogers and Anstis (1975) can not only explain our own reversed phi, reversed stereo and reversed Vernier offset effects but also the phenomenal phenomena effects described by Gregory and Heard (1983).

This is not to say that the motion, stereo and Vernier effects should be of the same *magnitude*. Anstis and Rogers (1975) reported that the spatial limit at which the reversed phi effect disappeared was ∼10 arc min). This is larger than the limit for the equivalent reversed stereo effects ∼6.5 arc min (Figure 7(b)) and larger still than the limit for the reversed Vernier effects ∼3.5 arc min (Figure 9(b)). Gregory and Heard (1983) made a similar statement about their phenomenal phenomena. How might our spatial-smoothing proposal account for these differences? As was pointed earlier, the spatial limit for seeing reversed phi between the original image and its displaced, contrast-reversed version was *larger* when the same stimuli were viewed in peripheral vision. This is consistent with the finding that the extent of spatial-smoothing is larger in peripheral retina. As a consequence, we propose that the amount of spatial-smoothing (low-pass filtering) is greater for the processing of motion stimuli than for binocular disparity which, in turn, is greater for the processing of Vernier alignment. This proposal is consistent with the suggestions made in the classic study of King-Smith and Kulikowski (1975).

## Kitaoka’s Colour-Dependent Fraser-Wilcox Patterns

As mentioned previously, Kitaoka (2006) has discussed the configurational coincidences between the motion, stereo and positional reversed effects described by Anstis and Rogers (1975) and the phenomenal phenomena described by Gregory and Heard (1983). Van Lier and Csathó (2006) have also noted the configurational coincidence between these effects and Cafe-Wall-like tilt illusions. The key feature common to all these effects is the presence of thin light or dark strips that are flanked on either side by regions that are either increasing or decreasing in luminance (Figure 3). In particular, when a thin light strip is flanked by a brightening region on its left and by a dimming region on its right, motion is perceived to the left.

Kitaoka (2014, 2017) has also drawn attention to the configurational coincidence between the stimuli used in his colour-dependent Fraser-Wilcox illusion and those used previously in A&R’s reversed motion, stereo and Vernier alignment studies. Given that the effects reported by A&R and G&H can be explained by a simple, spatial-smoothing model that effectively shifts the positions of the peaks, troughs and zero-crossings of the major contours (Figure 4), could a similar model predict the illusory motion seen in Kitaoka’s colour-dependent Fraser-Wilcox illusion? Note that Kitaoka has reported that the illusory motion effects are stronger when the colour-dependent Fraser-Wilcox patterns are viewed in peripheral vision (where there is greater spatial summation), just like the effects described by A&R and G&H.

There are, however, two important differences. First, the reversed motion, stereo and Vernier alignment studies involve the *comparison of image*s over time (in the case of reversed motion), between the eyes (in the case of reversed stereo) or spatially juxtaposed (in the case of reversed Vernier alignment). In contrast, only a single image is involved in the colour-dependent Fraser-Wilcox illusion. Under bright illumination conditions, such that the long-wavelength (red) flanking region (to the left of a light strip) is seen as getting *lighter* than the short-wavelength (purple) flanking region (to the right of a light strip), the perceived illusory motion is to the left. The second difference is that the colour-dependent Fraser-Wilcox illusion is almost certainly a colour effect—a grey-scale version produces little or no apparent motions (Kitaoka, 2014).

## Conclusions—Kitaoka’s Colour-Dependent Fraser-Wilcox Patterns

The luminance profiles used in A&R’s reversed motion effects are clearly very similar to those in Kitaoka’s colour-dependent Fraser-Wilcox figure with narrow light and dark strips flanking regions of homogeneous luminance. However, there are also important differences. It is possible that a difference in the latencies of different colour channels may play a significant role in creating the colour-dependent Fraser-Wilcox, but latency differences, by themselves, would not predict the perceived motion. However, the idea of differential latencies in different colour channels together with some sort of spatial smoothing to blur and alter the effective positions of the contours may provide a possible explanation of Kitaoka’s effect. At present, this is only speculation.

## Notes

1 Figure 4 shows the displacement of the smoothed contours in the reversed direction during the first six stages of the dissolve. Although not shown, those smoothed contours continue to show displacements in the reversed direction through the remaining stages of the dissolve to the displaced negative image.

2 One of our reviewers pointed out that motion energy models predict and therefore can “explain” Anstis’s reversed apparent motion effect. However, this is only true because motion energy models themselves incorporate spatial smoothing. Attributing the reversed motion effects to motion energy models is not an explanation, but a description of what happens when these particular stimuli provide the input to a particular motion energy model. We suggest that the reason why motion energy models predict reversed apparent motion effects is because they incorporate spatial smoothing. As a consequence, the reversed apparent motion effects, although consistent with the predictions of motion energy models, do not provide support for any particular motion model. If the reversed apparent motion contours are spatially smoothed, either optically or neurally, the contours themselves displace in the reversed direction (Figure 4), which means that *any* realistic motion model will signal the reversed motion.

3 In Figure 13(a) to (c), the black arrows on the left show that the peak of smoothed profile of the upper image (in green) is displaced to the *right* compared with the corresponding trough of the lower image (in red). The black arrows on the right show that that the trough of smoothed profile of the upper image (in green) is also displaced to the *right* compared with the corresponding peak of the lower image (in red).
